# Supplementary material for: Swelling, Rupture and Endosomal Escape of Biological Nanoparticles Per Se and Those Fused with Liposomes in Acidic Environment
Source: Pharmaceutics. 2024 May 16;16(5):667. doi: 10.3390/pharmaceutics16050667 (PMC11126099; doi:10.3390/pharmaceutics16050667)
Supplement: Supplementary file 1 [file pharmaceutics-16-00667-s001.zip › pharmaceutics-2921563-supplementary.pdf]

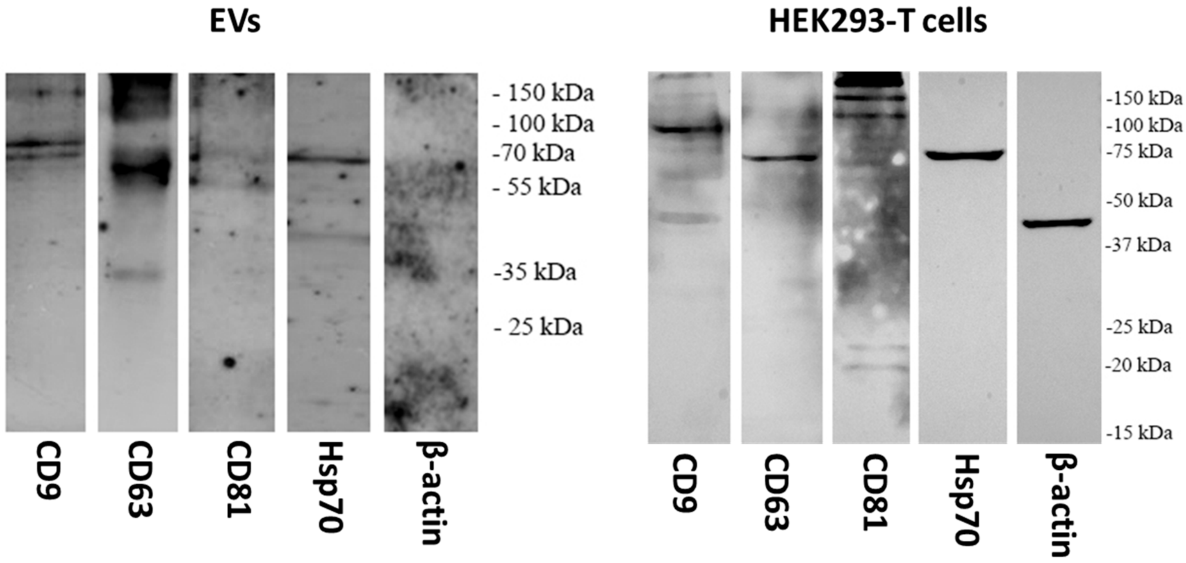

Figure S1. Protein expression analysis. Analysis of EVs-specific protein biomarkers (CD9, CD63, CD81, Hsp70) and β-actin as housekeeping control in isolated EVs and HEK293-T cells.
